# Pathogenicity of *Trichoderma afroharzianum* in Cereal Crops

**DOI:** 10.3390/pathogens12070936

**Published:** 2023-07-14

**Authors:** Annette Pfordt, Peet Gaumann, Andreas von Tiedemann

**Affiliations:** Institut of Plant Pathology and Crop Protection, Georg-August University Goettingen, Grisebachstrasse 6, 37077 Goettingen, Germany

**Keywords:** *Trichoderma*, maize, ear rot, host range

## Abstract

Species of the genus *Trichoderma* occur ubiquitously in soils, on plant roots and in decaying plant residues. Due to its competitiveness and mycoparasitic potential against other microorganisms, particular strains of *Trichoderma* spp. are used in agriculture as biocontrol agents against plant pathogens. However, *Trichoderma afroharzianum* has been recently reported as a pathogen causing ear rot disease on maize in Germany, France and Italy, leading to massive infections on maize cobs. This raised the question, whether and to what extent *Trichoderma* spp. can infect cereal crops other than maize and cause disease symptoms and yield losses. To address this question, two varieties of wheat, barley and sorghum were grown in the greenhouse and artificially inoculated with *T. afroharzianum* by both spray and point inoculation at the time of flowering. Disease severity was scored weekly, and thousand-kernel weight and colonization rate were determined after harvest. As early as 14 days after inoculation, the first visual symptoms appeared on wheat and barley as tan or brown discoloration of the base of a floret within the spikelets. After spray inoculation, clear discolorations of the entire ear were seen, while point inoculation only showed symptoms at the injection site and above. No visible symptoms were observed on sorghum millet. The colonization rate on wheat and barley grains was significantly increased compared to the control, while thousand-kernel weights (TKWs) were significantly reduced. No differences in colonization rate and TKW compared to the control were observed in sorghum. This is the first report of *Trichoderma afroharzianum* infecting wheat and barley, causing disease symptoms and significantly reducing thousand-kernel weights.

## 1. Introduction

Cereals belong to the family of Poaceae and are broadly used as a staple food for humans and livestock around the world. Among the most important cereals are wheat, barley and sorghum, which are grown and consumed globally. Wheat is one of the most widely grown cereals, and provides food for a large part of the world’s population. Global wheat production is estimated at 772 million tons in 2021 [1]. Barley is another important cereal used as animal feed and for human consumption; while wheat and barley are mainly produced under moderate climate conditions, sorghum is adapted to warmer climates and is one of the most important crops for human consumption and animal feed in tropical and subtropical regions. In 2018, sorghum was the world’s fifth largest crop, after wheat, corn, rice and barley [1]. 

Increases in the cultivation of cereals within corn rotations, combined with higher summer temperatures and increasing drought, have increased problems from infections with fungal pathogens [2,3]. *Trichoderma* spp. are filamentous fungi and are found worldwide in soils, on plant roots, on decaying plant debris and on wood. They are considered competent substrate colonizers and antagonists of other microorganisms, and are therefore used for biological control of plant diseases worldwide [4,5,6]. *Trichoderma* spp. may promote plant health by either directly countering pathogens through mycoparasitism, competitive inhibition and antibiosis, or indirectly via enhanced plant growth and stimulating systemic plant defenses [7,8]. *Trichoderma* spp. showed positive effects on plant growth and suppression of important cereal diseases. It has been shown to be effective in controlling Fusarium head blight, Fusarium crown rot and damping off by producing enzymes and secondary metabolites that inhibit the growth and reduce the levels of mycotoxins in wheat grains [9]. Thus, it had positive effects on suppressing leaf diseases such as tan spot disease caused by *Pyrenophora tritici-repentis* [10], and rust diseases caused by Puccinia spp. on wheat and barley [11,12,13]. Manzar et al. [14] indicated that *T. asperellum* is a potential biocontrol agent for managing anthracnose in sorghum. In addition, the root colonization promotes root growth and enhances nutrient uptake, which can improve the overall health and productivity of cereal crops [13,15,16].

Though widely considered beneficial, members of this fungal genus have been reported to be pathogenic on commercially important plants. In 2020, *Trichoderma afroharzianum* was reported for the first time in Europe as an ear rot disease in maize [17]. In this report, *T. afroharzianum* ear rot was characterized by massive production of gray-green spores in the interkernel regions and on the outer surface of the husks, causing a significant reduction of dry matter content and premature germination of kernels [17]. Since then, the pathogen has also been reported in France and Italy [18]. 

The recent spread of Trichoderma ear rot in maize raised the question of whether *T. afroharzianum* can also infect other cereal species which are commonly grown in crop rotation cycles with maize. Therefore, barley (*Hordeum vulgare*), wheat (*Triticum aestivum*) and sorghum (*Sorghum bicolor*) were cultivated in the greenhouse and inoculated at the time point of flowering with a mixture of pathogenic *T. afroharzianum* strains. Visual disease symptoms were assessed weekly, and thousand-kernel weight and colonization rate were determined after harvest.

## 2. Materials and Methods

### 2.1. Fungal Cultivation and Inoculum Preparation

*Trichoderma* strains were collected from naturally infected silage and grain maize in Germany in 2018 and 2019 (Table 1) [17]. Spore suspension was produced as described by Pfordt et al. [17], and diluted to 10^6^ conidia/mL. Tri1, Tri2 and Tri3 were mixed in the same ratio to prepare a mixed isolate (TriMix). Plants were inoculated with the pathogenic TriMix isolate and the apathogenic *T. afroharzianum* type strain CBS 124620 from the Westerdiijk Institute (CBS, Utrecht, The Netherlands). Control plants were inoculated with water (control). 

### 2.2. Plant Cultivation and Inoculation Procedure

Two varieties of wheat (*Triticum aestivum*), barley (*Hordeum vulgare)* and sorghum (*Sorghum bicolor*) were chosen for pathogenicity testing in the greenhouse. One winter wheat variety and one spring wheat variety, one spring malt barley and one winter barley and two sorghum varieties (silo and grain type) were used. 

**Figure 1:**
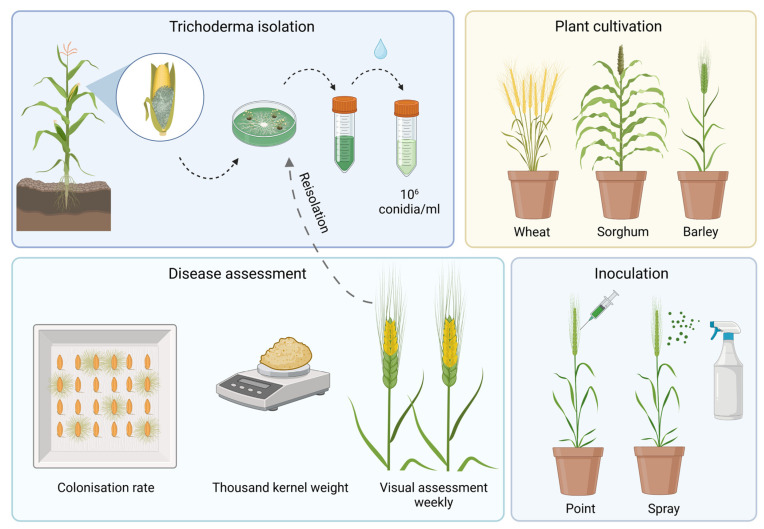
Method of pathogenicity testing with *T. afroharzianum* in wheat, sorghum and barley (created with Biorender.com).

Kernels of winter wheat and winter barley were vernalized for seven weeks at 4 °C, and planted in 7 cm diameter pots filled with a mixture of potting soil, sand and compost (3 × 1 × 3). Pots were placed in the greenhouse at 22 °C and subjected to a seasonal light cycle. Ten plants in two repetitions were inoculated with *T. afroharzianum*, and five plants per repetition were inoculated with sterile water, which served as control. Inoculation was conducted with two different inoculation methods at full anthesis. Point inoculation was carried out by syringe injecting 25 μL of spore suspension into the center of two florets. Spray inoculation was conducted by spraying 2 mL spore suspension from two sides on cereal heads (Figure 1). Ears were covered with plastic bags for 48 h post inoculation.

### 2.3. Disease Assessment

Severity of infection was visually scored weekly as percentage (0–100%) of diseased ear tissue. After harvest, kernels were counted, weighed and the weight extrapolated to 1000 grains to calculate thousand-kernel weight. To investigate the colonization rate, harvested seeds were surface disinfected for ten minutes with 0.25% silver nitrate, and placed on filter paper in growth chambers under sterile conditions. After 3 days, kernels with *Trichoderma* outgrowth were counted, and colonization rate was assessed by the number of infected kernels divided by the total number of analyzed kernels (Figure 1).

### 2.4. Germination Rate in Pots

Twenty seeds per treatment of previously inoculated grains were planted in pots in the greenhouse. Seeds were surface disinfected for ten minutes with 0.25% silver nitrate, and planted in 4 cm diameter pots filled with a mixture of potting soil, sand and compost. Pots were placed in the greenhouse at 22 °C with a 12 h day/night light cycle and watered as needed. Four weeks after planting, the emergence rate was assessed.

### 2.5. Statistical Analysis

Statistical analysis was conducted using STATISTICA version 13 (StatSoft GmbH, Hamburg, Germany). Differences between means of colonization rate and thousand-kernel weight were analyzed using parametric ANOVA by 5% probability. Analysis of variance (ANOVA) was carried out by Tukey’s HSD test at 5% probability. 

## 3. Results

### 3.1. Visual Disease Symptoms

**Figure 2:**
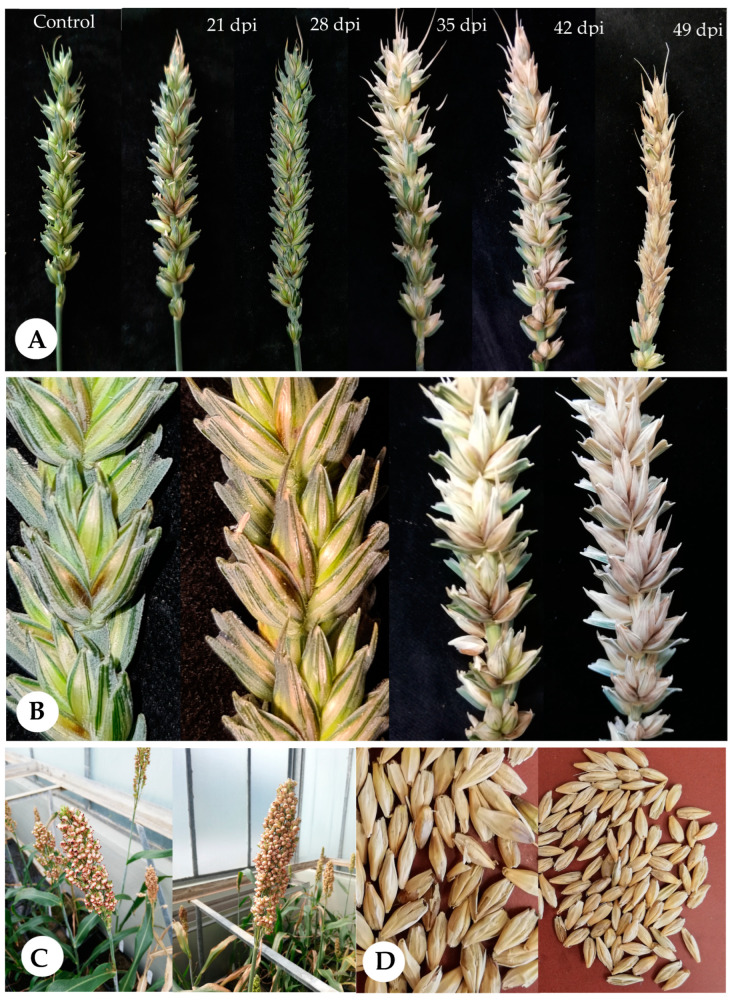
Disease symptoms in spring wheat at 21, 28, 35, 42 and 49 days post inoculation with pathogenic *T. afroharzianum* isolates (TriMix) (**A**), tan and brown discolorations at the base of a spring wheat floret at 28 days after inoculation with TriMix (**B**), lacking disease symptoms in inoculated sorghum varieties (**C**), and brownish discolorations and shriveled kernels of barley after inoculation with pathogenic TriMix at harvest (**D**).

First symptoms of Trichoderma ear rot occurred two weeks after inoculation. Distinct symptoms were observed as tan or brown discoloration of the base of a floret within the spikelets. Later, the base of the infected spikelets and parts of the rachis developed a dark brown color. The browning started at the injection site around the inoculated spikelet and spread up the ear until all spikelets above the infection showed discolorations. After spray inoculation, browning started at several spikelet’s on the whole ear and merged until the whole ear was discolored. As the infection progressed, infected spikelets bleached to become light tan. Infected kernels became shriveled and chalky, and kernels were light in weight (Figure 2A,B). Symptoms appeared similar in both varieties. 

Symptoms in barley were much less distinct. The brownish discoloration occurred three weeks after inoculation at the site of point inoculation. *Trichoderma* infection appeared as premature bleaching of individual or several spikelets, with tan discolorations at the base. The seeds in blighted heads did not fill properly, and appeared shriveled and bleached (Figure 2C). 

No visible disease symptoms were observed in sorghum (Figure 2D).

**Figure 3:**
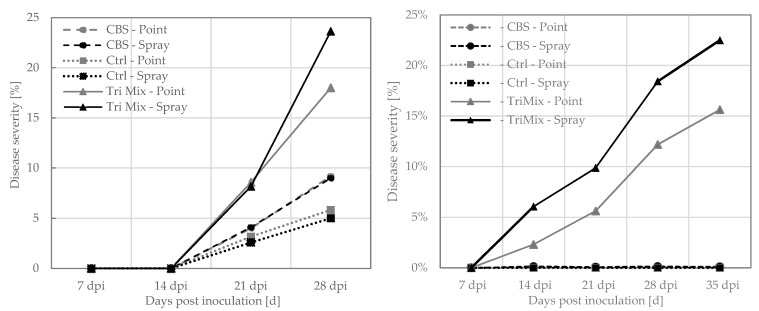
Disease severity (%) of diseased ear tissue at 7, 14, 21, 28 and 35 days post inoculation (dpi) of winter wheat (**left**) and spring wheat (**right**) after spray and point inoculation with pathogenic TriMix, apathogenic CBS 124620 isolate and water as control.

The first disease symptoms on spring wheat were observed 14 days after inoculation with TriMix, while disease symptoms on winter wheat occurred later, after 21 days (Figure 3). No symptoms were visible on control plants and type strain-inoculated spring wheat plants; however, slight symptoms were observed in winter wheat. Symptoms on wheat quickly developed to progressive bleaching of spikes and spikelets until 35 dpi (Figure 3). Later, the further disease progress could no longer be followed visually because heads turned yellow due to repining. Disease severity was highest after spray inoculation with TriMix, leading to more than 20% in both wheat varieties. Point inoculation with syringe lead to more than 15% disease severity in both varieties. Both TriMix treatments showed significantly higher disease levels than water-inoculated control plants. However, no disease symptoms were observed after inoculation with the CBS 124620 strain. 

Winter barley showed first disease symptoms 21 days after point inoculation with TriMix. Infection led to 32% of disease severity at 28 dpi. No visible symptoms were observed after spray inoculation in both varieties. Neither the water-inoculated control plants nor CBS 124620-inoculated plants showed any disease symptoms after inoculation with both methods. 

### 3.2. Colonization Rate and Thousand-Kernel Weight

**Figure 4:**
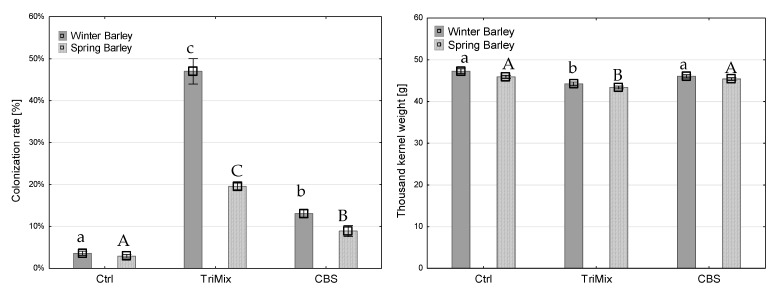
Colonization rate (%) (**left**) and thousand-kernel weight (g) (**right**) of winter and spring barley varieties after inoculation with water (Ctrl), pathogenic TriMix and apathogenic CBS 124620 isolate (means of both inoculation methods). Different letters indicate significant differences between treatments (Tukey’s test, *p* ≤ 0.05). Error bars represent standard error.

**Figure 5:**
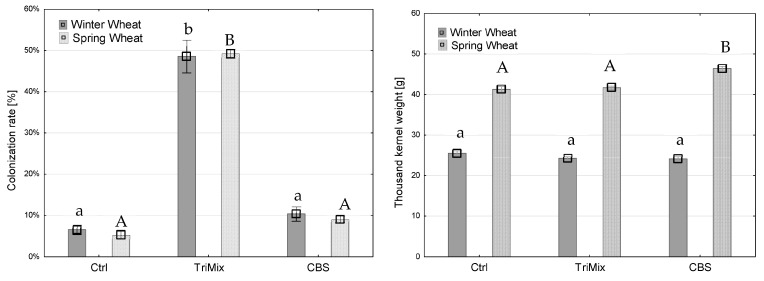
Colonization rate (%) (**left**) and thousand-kernel weight (g) (**right**) of winter and spring wheat varieties after inoculation with water (Ctrl), pathogenic TriMix and apathogenic CBS 124620 isolate (means of both inoculation methods). Different letters indicate significant differences between treatments (Tukey’s test, *p* ≤ 0.05). Error bars represent standard error.

After maturity, whole ears were harvested, threshed, weighed and thousand-kernel weight (TKW) was calculated. Afterwards, one hundred kernels were placed on filter paper and the colonization rate was assessed. The colonization rate after TriMix inoculation was significantly higher in both wheat and barley varieties than water and CBS 124620-inoculated plants. The colonization rate in winter barley after TriMix inoculation was the highest (47% of infected kernels), followed by spring barley with 20% (Figure 4). Inoculation with TriMix resulted in significantly higher colonization rates, and a significant reduction in TKW in winter and spring barley compared to water and CBS-inoculated plants. Strain CBS 124620 also led to a slight colonization of kernels of winter wheat; however, it did not differ significantly from control plants. TKW was significantly reduced after TriMix inoculation in both wheat varieties in comparison to water-inoculated control plants. Both wheat varieties showed similarly high colonization rates, with 48.6% and 49.3% of infected kernels after TriMix inoculation, which was significantly higher than CBS 124620 and water-inoculated plants (Figure 5). Thousand-kernel weight was not affected by TriMix inoculation overall for both inoculation methods. However, when the two inoculation methods are considered separately, it is shown that point inoculation resulted in a significant reduction in TKW in both varieties (Table 2). 

**Figure 6:**
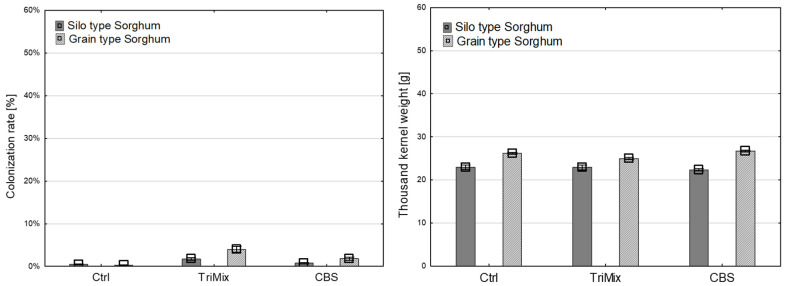
Colonization rate (%) (**left**) and thousand-kernel weight (g) (**right**) of silo type and grain type varieties of sorghum after inoculation with water (Ctrl), pathogenic TriMix and apathogenic CBS 124620 isolate (means of both inoculation methods). Error bars represent standard error.

After artificial inoculation in sorghum, neither colonization rate nor TKW was affected by the inoculation of pathogenic TriMix (Figure 6). 

There were significant differences found in the colonization rate and the TKW in relation to the inoculation method used, which was consistent for barley, wheat and sorghum varieties (Table 2). Inoculation with TriMix resulted in significantly higher colonization rates and a significant reduction in TKW in barley after spray inoculation, while point inoculation showed no significant differences in TKW compared to control plants (Table 2). Spray inoculation led to a higher colonization rate of 36.4% than point inoculation of 30.2%. Both varieties of wheat were highly infected with a 48.6% and 49.3% colonization rate, respectively. Similar to barley, spray inoculation led to a higher colonization rate (59.3%) than point inoculation (38.4%). However, point inoculation significantly reduced TKW in comparison to water-inoculated control plants. 

No significant differences were observed between the inoculation methods in sorghum after TriMix inoculation (Table 2).

### 3.3. Germination Rate

**Figure 7:**
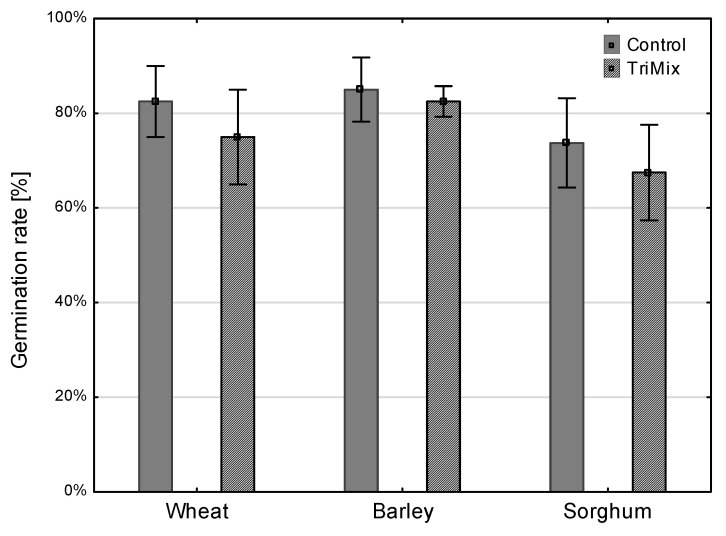
Germination rate (%) of wheat, barley and sorghum after inoculation with TriMix and water (control). Error bars represent standard error.

Germination rates in different crops were reduced after TriMix infection; however, no significant differences could be analyzed due to the low treatment number (Figure 7). 

## 4. Discussion

The results of the present study demonstrate that *T. afroharzianum* can infect wheat and barley, and causes visual disease symptoms as well as a reduction in TKW after artificial inoculation in the greenhouse. This is the first report describing *T. afroharzianum* as an ear-infecting pathogen in wheat and barley. Symptoms of infection were determined as a tan and brownish discoloration of spikelets, which spreads and coalesces until the entirety of the kernels are discolored. Finally, grains become shriveled and bleached. Apparently, the water and nutrient supply are cut off, both reducing kernel size and resulting in the shriveling of kernels [19]. These symptoms can be confused with extended symptoms of other wheat and barley diseases, such as Fusarium head blight, spot blotch or kernel smudge. This could be the reason the disease has not yet been described in the field. 

The severity of visual disease symptoms, in general, corresponds with colonization rate. High disease severity in wheat and barley after spray inoculation (25%) led to high colonization rates (50%), in contrast to low symptom severity after point inoculation (15%), which resulted in lower colonization rates (35%). However, colonization rates were higher than visual disease severity, indicating that more kernels were infected without showing symptoms. Moreover, no symptoms were observed in winter barley, although assessment of colonization rate showed 19.6% infected kernels. Visual ratings tended to underestimate the actual disease severity, probably because infected seeds partially appeared symptomless. 

Two inoculation methods were used in these investigations to study the ability of *Trichoderma* to infect and spread within the ears of cereal crops [19]. Both inoculation methods led to severe infections; however, point inoculation caused visible symptoms only at the site of inoculation and above, while spray inoculation led to severe infections on the whole ear. Point inoculation is a useful technique to investigate fungal growth and spread from the initial inoculation point within the head. In contrast, spray inoculation is mainly used to study the ability of the pathogen to initially infect the tissue without previous injuries [20]. The point inoculation method injects conidia via syringe directly inside a floret, which not only causes an artificial wound for fungal entry, but also provides optimal conditions for fungal colonization in the spike tissues. This inoculation method does not determine the fungal ability to cause initial infection, but provides information about the ability of the fungus to spread inside the ear between the florets [21]. In contrast, the spray inoculation with conidial suspensions, without wounding spike tissues, can assess the ability of the fungus to cause initial infection similar to what is happening under natural infection conditions in the field [22]. This study successfully demonstrates that *T. afroharzianum* can initially infect wheat and barley after spray inoculation, and spread between the florets. The results provide evidence that *T. afroharzianum* can infect cereal plant heads under controlled conditions and produce a head blight-like type of disease. It remains to be investigated whether this disease also occurs naturally under field conditions. 

Although, visual symptoms and colonization rates were higher after spray inoculation, point inoculation reduced the TKW in wheat and barley. The reduced kernel size may result from premature ripening, apparently induced by the shortage of water and nutrient supply due to a cut-off of transport vessels in the point-inoculated florets, as is known from Fusarium head blight [19]. It is not yet known how *Trichoderma* enters the florets and infects the interior floret tissue after spray inoculation, and how this happens under natural field conditions. It can be assumed that Trichoderma, similar to *Fusarium,* either directly penetrates the epidermal tissue of the inner palea and lemma, enters via natural crevices by hyphal growth or uses the anthers as primary tissue for the initiation of colonization [19]. 

*Trichoderma* species live endophytically in soils of all ecosystems and different agroclimatic zones [23,24]. Their rapid growth, the ability to parasitize other fungi, their high adaptability and competitiveness have rendered them worldwide importance as microorganisms in biological plant protection and biostimulant products [25,26]. They are widely used to control fungal diseases and promote plant growth in cereal crops such as wheat and barley [16,27,28]. Whether the use of biological fungicides and biostimulants poses a risk to cereal crops cannot yet be determined, since the routes of natural infection of *T. afroharzianum* in the field have not yet been adequately explored. Those biocontrol products can be applied to soil as liquid or granulate, foliar sprayed per handheld, tractor or even through irrigation water. However, our results suggest that especially spray inoculation in cereal cultivation caused infections of *T. afroharzianum* in wheat and barley, which resulted in high colonization rates and a reduction in TKW. We conclude that if spores of pathogenic *Trichoderma* species become dispersed on the spikes during flowering, the fungus can initially infect and spread in the cereal spikes. 

Overall, the results show that cereals such as wheat and barley are potential hosts enabling or even enhancing the survival and spread of *Trichoderma* in cereal crop rotations with maize. This risky situation is comparable to Fusarium ear rot, which can infect both maize and wheat via plant residues that represent a source of inoculum, and greatly increase the risk of disease [20,29]. 

This is the first report describing *T. afroharzianum* as a causal agent in wheat and barley leading to visible disease symptoms and reduction in TKW. The information provided in this work is important to better understand the epidemiology and disease cycle of *T. afroharzianum* within arable cropping systems comprising small grain cereals and maize. Hence, the development of management practices to reduce the amount of inoculum transmitted by crop residues also appears to be a relevant measure for *Trichoderma* epidemics. Further research is necessary to monitor whether pathogenic strains of *Trichoderma* from maize are in fact infecting wheat or barley under field conditions, and to explore under which conditions such host leaps may occur. 

## Figures and Tables

**Table 1:** Origin of strains used in this study.

Species	Isolate	Origin	Host
*T. afroharzianum*	CBS 124620 ^1^	Type strain	
*T. afroharzianum*	TriMix		
	-Tri1	Croix de Pardie	Maize
	-Tri2	Künzing	Maize
	-Tri3	Pocking	Maize

^1^ Westerdijk Fungalbio Diversity Insitute, CBS, Utrecht, The Netherlands.

**Table 2:** Colonization rate and thousand-kernel weight (TKW) of barley, wheat and sorghum after spray and point inoculation with pathogenic TriMix and water (control).

Crop	Method	Treatment	Colonization Rate (%)	TKW (g)
Barley	Spray	TriMix	36.4 a	43.0 a
		Control	4.8 b	47.9 b
	Point	TriMix	30.2 a	44.7 a
		Control	1.8 b	45.3 a
Wheat	Spray	TriMix	59.5 a	33.1 a
		Control	4.0 b	33.7 a
	Point	TriMix	38.4 a	33.4 a
		Control	7.7 b	34.8 a
Sorghum	Spray	TriMix	2.8 a	22.3 a
		Control	0.5 a	24.0 a
	Point	TriMix	3.0 a	25.5 a
		Control	0.3 a	25.1 a

Different letters indicate significant differences between pathogenic TriMix and Control (α ≤ 0.05, Tukey’s test).

## Data Availability

Not applicable.
